# Relief of post-stroke spasticity with acute vibrotactile stimulation: controlled crossover study of muscle and skin stimulus methods

**DOI:** 10.3389/fnhum.2023.1206027

**Published:** 2023-08-29

**Authors:** Caitlyn Seim, Bingxian Chen, Chuzhang Han, David Vacek, Laura Song Wu, Maarten Lansberg, Allison Okamura

**Affiliations:** ^1^Stanford University Department of Mechanical Engineering, Stanford, CA, United States; ^2^Stanford University Department of Bioengineering, Stanford, CA, United States; ^3^Stanford University Department of Neurology and Neurological Sciences, Stanford, CA, United States

**Keywords:** muscle spasticity, vibration, stimulation, stroke, stroke rehabilitation

## Abstract

**Background:**

Prior work suggests that vibratory stimulation can reduce spasticity and hypertonia. It is unknown which of three predominant approaches (stimulation of the spastic muscle, antagonist muscle, or cutaneous regions) most reduces these symptoms.

**Objective:**

Determine which vibrotactile stimulation approach is most effective at reducing spastic hypertonia among post-stroke patients.

**Methods:**

Sham-controlled crossover study with random assignment of condition order in fourteen patients with post-stroke hand spasticity. All patients were studied in four conditions over four visits: three stimulation conditions and a sham control. The primary outcome measure was the Modified Ashworth Scale, and the secondary outcome measure was the Modified Tardieu Scale measured manually and using 3D motion capture. For each condition, measures of spastic hypertonia were taken at four time points: baseline, during stimulation, after stimulation was removed, and after a gripping exercise.

**Results:**

A clinically meaningful difference in spastic hypertonia was found during and after cutaneous stimulation of the hand. Modified Ashworth and Modified Tardieu scores were reduced by a median of 1.1 (SD = 0.84, *p* = 0.001) and 0.75 (SD = 0.65, *p* = 0.003), respectively, during cutaneous stimulation, and by 1.25 (SD = 0.94, *p* = 0.001) and 0.71 (SD = 0.67, *p* = 0.003), respectively, at 15 min after cutaneous stimulation. Symptom reductions with spastic muscle stimulation and antagonist muscle stimulation were non-zero but not significant. There was no change with sham stimulation.

**Conclusions:**

Cutaneous vibrotactile stimulation of the hand provides significant reductions in spastic hypertonia, compared to muscle stimulation.

**Clinical trial registration:**

www.ClinicalTrials.gov, identifier: NCT03814889.

## 1. Introduction

Spastic hypertonia is a common complication after central nervous system injury affecting 30–40% of people with impaired limb function after stroke (Watkins et al., 2002; Dajpratham et al., 2009). With the population living with chronic stroke in the US expected to double from 2010 to 2050, spastic hypertonia is also a growing problem (Ovbiagele et al., 2013; Mozaffarian et al., 2016).

Spastic hypertonia (hyperreflexia called “spasticity” and elevated involuntary muscle tone) can cause patient discomfort, impair sleep, and make activities like dressing the upper body difficult (Patel et al., 2019). Spastic hypertonia can increase when the limb is in use or when medication treatments, like Botulinum toxin, are wearing off (Sommerfeld et al., 2004). Over time, spastic hypertonia can impair cleaning the hand, cause skin breakdown, and lead to contractures that permanently limit joint mobility (Thompson et al., 2005).

For many patients, current treatment options are inadequate. The most common therapies for spastic hypertonia are intramuscular injection of botulinum neurotoxin type A (BTX-A) and oral antispasmotics like Baclofen. BTX-A injections are typically repeated every three months, with 83–88% of patients reporting relapse of symptoms of spastic hypertonia between injections (Jacinto et al., 2020; Comella et al., 2021). Moreover, treatment does not fully abolish symptoms, even at peak effect (Comella et al., 2021). Baclofen is associated with side effects such as drowsiness (Ertzgaard et al., 2017), and BTX-A is associated with side effects such as weakness (Yaraskavitch et al., 2008; Crowner et al., 2010). Splinting is used to immobilize and stretch the limb to prevent contractures, but does not relieve spastic hypertonia (Lannin and Herbert, 2003).

Research has provided promising preliminary evidence that vibratory or vibrotactile stimulation may help relieve spastic hypertonia symptoms (Ness and Field-Fote, 2009; Noma et al., 2009, 2012; Marconi et al., 2011; Murillo et al., 2011; Caliandro et al., 2012; Casale et al., 2014; Costantino et al., 2017). Vibration can provide strong afferent input through both muscle sensory receptors (Ia) (Roll et al., 1989) and cutaneous mechanoreceptors (Verrillo et al., 1969; Johansson and Vallbo, 1979). However, the dominant mechanism acting to provide spastic hypertonia relief is unknown. In this prior research, three approaches to afferent stimulation for spasticity relief have emerged, including, stimulation to (1) the spastic (agonist) muscle (Noma et al., 2009, 2012; Marconi et al., 2011; Caliandro et al., 2012; Costantino et al., 2017; Won and Park, 2018), (2) the antagonist to the spastic muscle (Hagbarth and Eklund, 1968; Ageranioti and Hayes, 1990; Binder et al., 2009; Cordo et al., 2009; Murillo et al., 2011; Casale et al., 2014), and (3) the skin (Levin and Hui-Chan, 1992; Dewald et al., 1996; Cho et al., 2013; Seim et al., 2021b). No prior work has tested these different approaches in the same participant group. To address this question, we performed a controlled experiment comparing all three approaches to vibration therapy and a sham in a group of patients with post-stroke spastic hypertonia. Spasticity symptoms were measured before, during, and after stimulation to provide data on the time course of symptom changes. We hypothesize that measures of spastic hypertonia will be reduced during stimulation (τ1−τ0) for all conditions. We expect there to be some reduction in spastic hypertonia in the control condition due to relaxation of the participant while sedentary.

## 2. Methods

We conducted a randomized sham-controlled crossover study of vibrotactile stimulation in patients with spasticity after stroke. This study was reviewed and approved by the Stanford University Institutional Review Board and was registered at ClinicalTrials.gov (Identifier: NCT03814889).

### 2.1. Participants

Fourteen individuals with post-stroke spastic hypertonia provided informed consent and participated in the study. Participants were recruited through local clinics and stroke support groups. The inclusion criteria were: history of stroke ≥ 6 months prior and spastic hypertonia measured at the proximal interphalangeal (PIP) and/or metacarpophalangeal (MCP) joints (score ≥ 1 on the Modified Ashworth Scale) when all fingers are moved simultaneously.

Participants who were taking antispasmodics were allowed to continue their medications. The exclusion criteria were: botulinum type A (BTX-A) injection within the last three months, contracture or injury limiting the PIP/MCP maximum passive range of motion, other conditions that affect movement (e.g., MS, ALS, Parkinson's).

### 2.2. Stimulation locations

In this work, we applied three methods of therapeutic vibratory stimulation and a sham stimulation:

Agonist muscle stimulation applied to an extrinsic finger flexor muscle [the flexor digitorum superficialis (FDS)] near its exposure on the middle forearm. Prior research applying stimulation to the spastic muscle suggests that this stimulation works by activating the Ia muscle spindles (Caliandro et al., 2012) which may cause decreased response to stretch (spasticity) from post-activation depression (Costantino et al., 2017), reduction of muscle tone via presynaptic or recurrent inhibition (Noma et al., 2012), or reduced muscle hyperexcitability through central Hebbian-based long-term potentiation of inhibitory tracts (Marconi et al., 2011; Caliandro et al., 2012).
Antagonist muscle stimulation applied to the extrinsic finger extensor muscle [the extensor digitorum (ED)]. Prior work applying stimulation to the spastic antagonist muscle suggests that this works through Ia muscle spindle activation, which induces reciprocal inhibition of the opposing (spastic) muscle (Ageranioti and Hayes, 1990).
Cutaneous stimulation applied to the fingertips. This zone was chosen for three reasons: (1) The fingertips are dense in cutaneous mechanoreceptors, including the Pacinian corpuscles which are the most responsive to vibratory input (Johansson and Vallbo, 1979). (2) The fingertips are one of the most isolated zones from nearby muscle on the upper limb. While the intrinsic hand muscles and tendons are present nearby, our stimulation was designed to be low amplitude to enhance localization at the fingertip skin. (3) Data from other work suggest that cutaneous input at the fingertips or volar hand not only can produce reflex inhibition in the forearm flexor muscles (Nielsen and Pierrot-Deseilligny, 1991), but may also facilitate co-activation in the extensor muscles (Kim et al., 2013).
Sham stimulation applied to the dorsal wrist.

### 2.3. Apparatus and stimulation settings

**Figure 1:**
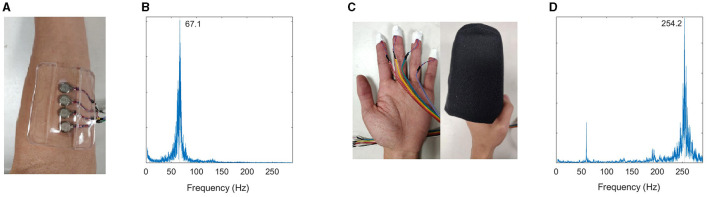
**(A)** Muscle stimulation setup. **(B)** Validation of muscle stimulation frequency measured using accelerometer. Muscle stimulation was tuned to the muscle afferent receptors. **(C)** Finger stimulation setup. The mitten in the image is pictured on an open hand, but during the study, it is applied in the hand's neutral position without stretching it open. **(D)** Validation of cutaneous stimulation frequency—tuned to the cutaneous afferent receptors.

Vibration was delivered using vibration motors (Precision Microdrives, Model #310-103 and #C08-005) connected to a circuit board via ribbon cable. During muscle stimulation conditions, four motors (Model #310-103) were attached to the affected arm over the target muscle using a silicone adhesive pad (Outus brand) (Figure 1A). The gel pad helps to prevent the vibration from spreading. The motors vibrate one at a time (500 ms), in a sequence with no pauses in between.

During fingertip stimulation, the motors (Model #C08-005) were attached to each finger of the affected hand using a small adhesive tape (Figure 1C). The motors vibrate in the same pattern described above. The fingers were then slipped into a foam mitten. The foam mitten slipped over the first two digits of the fingers while the hand remains curled, acting as a buffer to prevent the motors coming in contact with the palm. The hand is not stretched open while wearing or donning the mitten. The mitten is made from 1.25^′′^ thick foam on the dorsal side, 2.5^′′^ thick foam on the volar side, and enclosed with stretch cotton fabric.

#### 2.3.1. Frequency

A small, battery-powered circuit board ran the motors. During the trials, the circuit board was usually tucked inside the participant's sleeve. Our target frequency for muscle stimulation was between 70–90 Hz. Prior work used frequencies between 70–90 Hz to stimulate the Ia muscle sensory fibers of agonist and antagonist muscles (Siggelkow et al., 1999; Rosenkranz and Rothwell, 2003; Steyvers et al., 2003; Forner-Cordero et al., 2008; Marconi et al., 2008; Binder et al., 2009; Cordo et al., 2009, 2013). These receptors respond in a synchronous 1:1 rate to vibration stimuli up to 100–150 Hz (Roll et al., 1989; Park and Martin, 1993; Martin and Park, 1997). Contraction of the stimulated muscle increases the Ia discharge Our target frequency for skin stimulation was 250 Hz. The tactile mechanoreceptors are most responsive to vibration at 150–250 Hz (Pacinian corpuscles preferentially respond around 250 Hz) (Verrillo et al., 1969; Johansson and Vallbo, 1979).

#### 2.3.2. Amplitude

Because the aim of this work is to apply localized stimulation to a select muscle or skin region, amplitude settings were determined during system validation. We adjusted amplitude settings to provide localized stimulation and minimize transmission to other tissues. Increasing the amplitude of stimulation increases the transmission of vibration through tissue to reach more sensory receptors (Eklund and Hagbarth, 1966; O'Mara et al., 1988; Park and Martin, 1993; Kihlberg et al., 1995; Guang et al., 2018).

Vibration amplitude and frequency were measured for each apparatus using an accelerometer at 10 kHz (Kistler, Inc.). The amplitude and frequency were measured for one vibration motor within each apparatus while it lay on a table (Figures 1B, D). We also measured vibration at the target zone and adjacent regions when each apparatus was applied to the upper limb. Adjacent regions were measured to assess vibration transmission through the limb, and amplitude was adjusted to provide stimulation at the target zone while limiting stimulation elsewhere. For flexor (agonist) stimulation, the sites of interest were as follows: distal (1.5^′′^ from the motor along the FDS toward the hand), medial (at the boundary between FDP and extensor carpi ulnaris, 2.5^′′^ medial from stimulation site), lateral (at the brachioradialis, 2.5^′′^ medial from stimulation site), at the antagonist muscle [at the extensor digitorum (ED)]. For extensor stimulation, the sites of interest were as follows: distal (1.5^′′^ along the ED toward the hand), medial (at the boundary between FDP and extensor carpi ulnaris, 2.5^′′^ medial from stimulation site), lateral (at the brachioradialis, 2.5^′′^ medial from stimulation site), and antagonist (at the FDS). Frequencies of 67–70 Hz were achieved for muscle stimulation and 253–255 Hz for skin stimulation. Vibration amplitudes were low at non-target locations: ~0.18 mm at non-target muscle groups (vs. 0.61 mm at target muscles), and <0.08 mm at non-target areas during fingertip stimulation. Prior work applied vibrotactile stimulation of 0.5–2 mm (Marconi et al., 2011; Caliandro et al., 2012; Noma et al., 2012; Alashram et al., 2019), and amplitudes less than 0.3 mm have been reported to show nonsignificant effects on muscle spasticity vs. higher amplitudes (Seo et al., 2016).

### 2.4. Measures

The following measures were obtained to assess the response to vibratory stimulation.

The Modified Ashworth Scale (MAS) for the MCP and PIP joints was the primary outcome measure.
The Modified Tardieu scale (MTS) for the MCP and PIP joints served as a secondary measure of spastic hypertonia symptoms. MTS includes a rating and angle measure both indicating levels of spasticity. MTS focuses on the mechanical properties of the stretch reflex (spasticity): gain (rating) and catch angle (MTS angle). Stretch reflex properties are a “readily observable marker of spinal motoneuron excitability (McPherson et al., 2019).” We elected to use biomechanical measures like MTS (rather than electromyography, EMG) because they are functionally relevant, do not require calibration between subjects, and can be measured without signal disturbance during vibration. In addition, EMG and Hoffman waves are difficult to noninvasively capture in the forearm, especially of paretic patients (Rafiee et al., 2011). MTS angle is reported as the maximum passive range of motion (how far the joint can be extended at a very slow speed by an examiner) minus the catch angle (the angle a catch or clonus is found during a quick stretch by an examiner). A digital goniometer was used to measure joint angles.
The Isometric Force Pillow (IFP) (Seim et al., 2020) is an experimental tool used to measure involuntary grip force from the fingers. Flexor spastic hypertonia causes the fingers to squeeze and bend. This tool provides a holistic, quantitative value of this symptom by measuring the pressure exerted by the fingers when the person is relaxed.
Motion capture The MTS angle was also measured using 3D motion capture markers attached to each joint on the upper limb.

Circumstantial factors such as arousal, posture, and room temperature have a strong influence on both velocity-dependent spastic reflexes and hypertonia (Grillon and Zarifian, 1985). Factors that influence spastic hypertonia were controlled during the experiment to provide the most accurate data possible: Laboratory temperature was regulated at 72 degrees. Measures were recorded in a standardized position/posture for all participants. The measurement position is gravity-neutral—preventing any gravity assistance in voluntary motion or spastic hypertonia measures. If participants' wrists and arms did not rest in a gravity-neutral position with the wrist straight, the investigator or assistant held the arm in position. All participants were provided a 10-min rest period after arriving at the lab. Measures were administered in a predetermined order that recorded spastic hypertonia before voluntary range of motion tests (which could temporarily excite spastic hypertonia measures).

### 2.5. Experimental design

Each participant made four visits to the laboratory. At the first visit, participants were assigned a randomized order of the four conditions (flexor muscle stimulation, extensor muscle stimulation, fingertip stimulation, and control) using opaque envelopes. During each visit, the participant was administered one stimulation condition according to their assigned condition order.

During each session, measures were taken at four different time points: baseline (τ0), during the last 2 min of a 20-min stimulation period (τ1), 15 min after stimulation is removed (τ2), and after a final gripping exercise (τ3).

The visit procedures for each visit were as follows:

1. Rest and adjust (10 min). The visit begins with an adjustment period during which the participant reads or watches a video for 10 min.
2. Measures (τ0). All measures (time τ0) were performed on the affected limb in the following order: MAS, MTS, and IFP. These measures were performed in view of a motion capture system while the patient is seated and their arm is in the gravity-neutral position. Any discrepancy in arm position due to severe hypertonia was noted.
3. Stimulation condition while watching videos (20 min). The investigator helped the participant don the apparatus corresponding to the session's stimulation condition. The participant then was asked to rest (read or watch a video) during 20 min of wearing the apparatus. Participants were offered breaks as needed.
4. Measures (τ1). During the last 2 min of the stimulation period, the investigator recorded all measures again (time τ1). Measures at time τ1 represent the arm's state during stimulation, while receiving afferent input. Mechanisms such as postactivation depression and reciprocal inhibition are expected to be active only during stimulation. For the control condition, τ1 measures provide data on the arm's state after a period of relaxation with no (sham) stimulation.
5. Remove stimulation and watch videos (15 min). The investigator turned off and removed the stimulation apparatus. The participant then sat while watching a video.
6. Measures (τ2). After the 15-min period of rest, all measures (MAS, MTS, and IFP) were taken again (time τ2).
7. Gripping exercise. Following time τ2, participants were asked to make a tight fist and extend both arms for 10 s, three times, to increase excitatory drive to the hand.
8. Measures (τ3). Immediately after the gripping exercise, all measures were taken again (τ3) to determine the arm's response to excitatory drive after receiving stimulation.

### 2.6. Conditions

Participants were blinded to the different conditions and were not informed that there was a sham-control condition. Proctors were not involved in the development of the study protocol, conditions, or hypotheses. Proctors were taught to administer each condition, but were not informed of the rationale behind each condition.

The control condition (sham) was designed to be indistinguishable from the other conditions but provide no meaningful stimulation. In this condition, the silicone pad of motors was attached at the wrist of the affected arm. The circuit board was turned on, but the motors did not activate. There was no difference in sound to participants. A fan in the lab created a white noise at ~50 dB, which obscured the sound of the motors in all conditions. No participant reported not feeling the sham stimulation. For some participants, stimulation in the other conditions may be imperceptible

### 2.7. Data analysis

For each condition, participants' baseline values (τ0) were compared to values during stimulation (τ1). Wilcoxon Signed-Rank tests were performed on paired ordinal data (Modified Ashworth and Modified Tardieu scale ratings) and paired *t*-tests were performed on paired continuous variables (Modified Tardieu angle measures). Values after stimulation (τ2) were compared to baseline using the same tests. For a given condition, if a significant difference had been found during or after stimulation, values after the gripping exercises were also compared to baseline in order to examine if spastic hypertonia had returned to pre-stimulation levels. We also conducted a Friedman's test to assess whether baseline scores differed significantly between conditions. The alpha value was set at 0.05 and the Benjamini–Hochberg procedure was used to control for the false discovery rate. 3D motion capture values for catch angle and maximum passive range were the mean of two and three excursions, respectively.

### 2.8. Power analysis

We carried out power calculations using prior work which applied acute afferent/vibratory stimulation at the spastic agonist muscle (Noma et al., 2009), the antagonist muscle (Murillo et al., 2011), or the skin (Cho et al., 2013). Our sample size calculations were based on a two-sided paired *t*-test, which should have adequate power for the Wilcoxon Signed Rank test. For agonist stimulation, we expected the mean change on the Modified Ashworth scale to be −2.3 and the standard deviation to be 0.5 (Noma et al., 2009). We found that we can achieve 80% power with four samples. For antagonist stimulation, we expected the change to be −1.1 and the standard deviation to be 0.6 (Murillo et al., 2011). We found that we can achieve 80% power with six samples. For cutaneous stimulation, we expected the change to be −1.0 and the standard deviation to be 0.75 (Cho et al., 2013). We found that we can achieve 80% power with nine samples. We enrolled five additional participants to account for those who may become lost to follow-up, leading to the sample size of 14 participants. Use of the Modified Tardieu Scale is less common in the related literature, but we expected the test to be of the same sensitivity as Modified Ashworth Scale.

## 3. Results

The participant mean age was 60 years (33–75 years). The biological sex distribution was five females and nine males. The race and ethnicity distribution was: seven Asian, six Caucasian, and one Hispanic or Latino.

**Figure 2:**
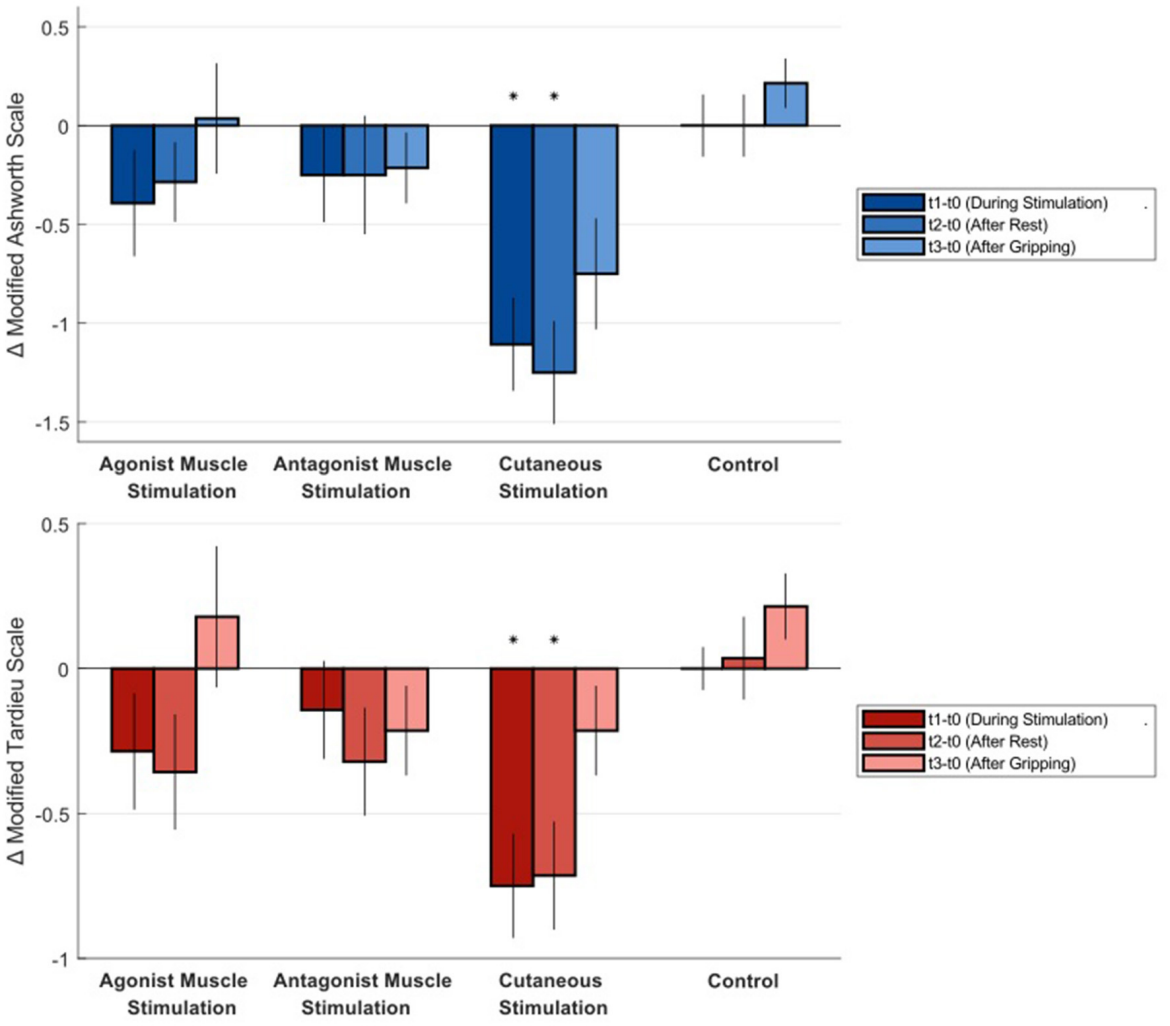
Average difference in Modified Ashworth Scale and Modified Tardieu Scale ratings from baseline at each time point for all conditions. Negative numbers represent a reduction in spastic hypertonia. Statistical significance is indicated by an asterisk. MAS ratings are presented on a scale of 0–5. Error bars show standard error.

Table 1 shows Modified Ashworth Scale ratings and Modified Tardieu values for all time points and all conditions. Figure 2 shows the difference from baseline at each time point for all conditions. Trends over all time points are also plotted in Supplementary Figures S1, S2. Only cutaneous simulation resulted in significant reductions in Modified Ashworth ratings (Mean change −1.11 at τ1, *p* = 0.001; −1.25 at τ2, *p* = 0.001; and −0.75 at τ3, *p* = 0.011), Modified Tardieu ratings (Mean change −0.75 at τ1, *p* = 0.003; −0.71 at τ2, *p* = 0.003; and −0.21 at τ3, *p* = 0.1), and Modified Tardieu angle (Mean change −12.68° at τ1, *p* = 0.002; −9.54° at τ2, *p* = 0.007). MAS ratings are presented on a scale of 0–5.

**Table 1:** Average Modified Ashworth Scale and Modified Tardieu values at each time point for all conditions. MAS ratings are presented on a scale of 0–5. SD is standard deviation. The score for each participant at a given time point was calculated by averaging their PIP and MCP values.

		**MAS rating**		**MTS rating**		**MAS angle**	
		**Avg**.	**SD**	**Avg**.	**SD**	**Avg**.	**SD**
Agonist muscle stimulation	Baseline (τ0)	3.04	1.26	2.57	1.05	51.5	15.73
	During stimulation (τ1)	2.64	1.44	2.29	1.06	45.82	19.7
	After rest (τ2)	2.75	1.05	2.21	0.96	46.57	18.96
	After gripping exercise (τ3)	3.07	1.03	2.75	0.82	48.32	17.24
Antagonist muscle stimulation	Baseline	2.61	0.85	2.32	0.79	55.18	15.19
	During stimulation	2.36	0.83	2.18	0.97	49.96	17.00
	After rest	2.36	0.85	2	0.87	44.82	14.77
	After gripping exercise	2.39	0.78	2.11	0.89	48.04	17.16
Finger cutaneous stimulation	Baseline	3.14	0.93	2.29	0.84	48.86	17.73
	During stimulation	2.04	1.06	1.54	0.81	36.18	18.42
	After rest	1.89	1.28	1.57	0.88	39.32	20.93
	After gripping exercise	2.39	1.07	2.07	0.88	42.07	19.08
Control	Baseline	2.64	0.95	2.11	0.78	42.75	14.21
	During stimulation	2.64	0.91	2.11	0.74	46.93	22.78
	After rest	2.64	0.79	2.14	0.69	46.46	16.58
	After gripping exercise	2.86	0.87	2.32	0.79	47.46	18.04

We conducted a Friedman's test to assess whether baseline spasticity scores differed significantly between conditions. For the MAS baseline values, the test showed no significant difference between conditions (*p* = 0.051, Friedman's statistic = 7.7786, *N* = 14). Similarly, for the MTS baseline scores, there was no statistically significant difference between conditions (*p* = 0.126, Friedman's statistic = 5.7214, *N* = 14).

The MTS angle was also measured using 3D motion capture. 3D motion capture data showed that the catch angle was measured at a mean joint movement speed of 132 degrees per second, and the passive range of motion was measured at a mean of 22 degrees per second. The motion capture measures of MTS angle are graphed alongside the manual measurements in the Supplementary material. Values using 3D motion capture were similar to values taken by hand with a digital goniometer for all conditions. Precision of measures taken by hand was also similar to 3D motion capture measures (average SD difference = 5°). The Isometric Force Pillow data is reported in the Supplementary material.

## 4. Discussion

There are multiple approaches to vibratory stimulation in the context of spastic hypertonia, and the work here is the first to compare multiple approaches in the same population. This comparison is important to provide more data on both the underlying mechanisms and optimal approach.

Results indicate that 20 min of skin stimulation, here applied to the fingertips, was associated with a significant reduction in measures of spastic hypertonia. Measures were still significantly different from baseline after stimulation was removed. Peripheral effects, like inhibitory postsynaptic potentials triggered by a volley of sensory input, would be expected to last only during stimulation (Dideriksen et al., 2015). This data thus supports to the idea that cutaneous stimulation could interact with mechanisms with longer time scales. Dewald et al. (1996) discussed a similar finding when using cutaneous electrical stimulation: They found reductions in spastic reflex torque that lasted 15 min after removal of stimulation. They discuss that synaptic effects like reciprocal and recurrent inhibition, as well as presynaptic inhibition are known to last less than a second after the stimulation input. They suggest that long-term potentiation (LTP) or depression (LTD) mediated via primary afferents at the dorsal horn could be at work. “*Given that we observe a reduction in stretch reflex responsiveness after cutaneous stimulation, LTP would presumably operate at a convergent inhibitory interneuron, while LTD would operate at a presynaptic excitatory neuron”* (Dewald et al., 1996).

Mean difference in Modified Ashworth Scale ratings of 1.1-1.25 points during and after cutaneous finger stimulation also surpass a clinically meaningful difference. The minimal clinically important difference (MCID) in Modified Ashworth for the upper limb is between 0.45 and 0.73 points (Chen et al., 2019).

Though not statistically significant, non-zero changes were also found during both conditions of muscle stimulation. The reason that muscle stimulation was not associated with a significant change in spastic hypertonia could be due to several factors. In prior work, the limb was sometimes in a stretched position e.g., Noma et al. (2009)—which keeps muscles long for increased Ia receptor discharge and keeps the hand loose for measurement. Muscle vibration thus may be best combined with splinting to keep the muscles in a stretched position—a potential avenue for future work. Prior work in muscle stimulation (including both whole body vibration and focal muscle vibration targeted to a specific muscle group) also likely activated multiple agonist and antagonist pathways partly due to the apparatus used in prior work. For example, if our focal stimulation apparatus applies 0.6 mm vibration that transmits ~0.18 mm to non-targets, prior work that applied 1 mm vibration or greater (Casale et al., 2014; Alashram et al., 2019), even using a rounded probe, could experience significant transmission to non targets (e.g., antagonists) (Guang et al., 2018). More than 0.3 mm has been reported to show significant effects in spastic hypertonia (Seo et al., 2016). Of course, stimulation at any muscle group will produce some effect in both agonist and antagonist circuits. Perhaps a higher amplitude of vibration would improve muscle stimulation for spastic hypertonia. Though such stimulation could not be isolated to a particular muscle group, the increased spread of higher-amplitude vibration could increase afferent discharge or muscle fiber recruitment.

The gripping exercise, adapted from the Jendrassik maneuver (Zehr and Stein, 1999), was intended to increase excitatory drive to the impaired limb. Though activities such as walking may provide more drive, our exercise appears to be a useful and accessible procedure. The data at τ3 serve as an effective addition to our repeated measurements in a given visit. Our hypothesis that measures of spastic hypertonia increase after an excitatory activity in all conditions was confirmed. Change in Modified Ashworth measures for the skin stimulation condition increased after the gripping exercise, but did remain significantly below baseline. This finding provides more support that muscle tone inhibition in this condition is not solely supported by an inflow of inhibitory postsynaptic potentials.

Our hypothesis that measures of spastic hypertonia would decrease during the control condition was not confirmed. Measures during the control condition were more consistent that we expected—enabling a clear comparison between conditions. We understood that participants may exhibit some relaxation (leading to decreased spastic hypertonia) after sitting and watching videos during the visit. We provided an adjustment period after their arrival to the lab for the explicit purpose of allowing the participant to stabilize, which appears to have been effective.

The results here inform the development and future research into vibratory and vibrotactile stimulation for spastic hypertonia relief. If a wearable device is created, this stimulation could be applied at intervals throughout the day to help relieve elevated spastic hypertonia during therapy or activities, in the morning, or as-needed. It could be used to help relax the limb for splinting, or between pharmaceutical doses (e.g., Botox or Baclofen). Stimulation is also accessible to those with limited residual motor function who cannot perform exercises.

## 5. Limitations

No non-invasive apparatus can fully exclude activation of non-target muscles and overlying skin. The stimulation conditions used here were designed and validated to provide minimal stimulation of the non-target zones. In addition to amplitude choices, the stimulation's *location* and *frequency* support the difference in stimulation methods. The skin of the forearm contains significantly fewer cutaneous receptors than the fingertips. In fact, even individuals without stroke are unable to perceive vibrations at up to 1.5 g amplitude on the arm (Seim et al., 2021a). Some cutaneous activation will still occur during the muscle stimulation conditions; however, this is further mitigated by the use of a 70 Hz stimulation frequency, which is 1/4th the frequency at which Pacinian corpuscles preferentially respond to vibration [250 Hz (Johansson and Vallbo, 1979)]. In contrast, 70 Hz elicits near the peak response from Ia muscle spindles (Roll et al., 1989). Finally, this work focused on short term effects and patients in the chronic stage of stroke. Future work should further evaluate the potential clinical impacts of stimulation approaches long term.

## 6. Conclusions

Cutaneous vibrotactile stimulation of the hand provides significant reductions in spastic hypertonia, compared to muscle stimulation.

## Data availability statement

The datasets presented in this article are not readily available because patient data cannot be shared publicly. Requests to access the datasets should be directed to cseim@stanford.edu.

## Ethics statement

The studies involving humans were approved by Stanford University Institutional Review Board. The studies were conducted in accordance with the local legislation and institutional requirements. The participants provided their written informed consent to participate in this study.

## Author contributions

BC and DV administered the study and supervised data entry. CS, CH, and LW engineered study devices. CS, AO, and ML designed the study protocol, analysis, and reporting. All authors contributed to the article and approved the submitted version.
