# Supplementary material for: Relief of post-stroke spasticity with acute vibrotactile stimulation: controlled crossover study of muscle and skin stimulus methods
Source: Front Hum Neurosci. 2023 Aug 29;17:1206027. doi: 10.3389/fnhum.2023.1206027 (PMC10497102; doi:10.3389/fnhum.2023.1206027)
Supplement: Supplementary file 1 [file Data_Sheet_1.PDF]

Supplementary Materials: "Relief of Post-Stroke Spasticity With Acute Vibrotactile Stimulation: Controlled Crossover Study of Muscle and Skin Stimulus Methods"

1 POWER CALCULATION

Table S1 displays the data from related work used in our power calculation.

Author	# of Participants	Approach	Duration	Measure	Avg. Δ	SD
Noma et al. [3]	14	Agonist stimulation	5 minutes	MAS	-2.3*	0.5
Murillo et al. [2]	19	Antagonist stimulation	10 minutes	MAS	-1.1*	0.6
Cho et al. [1]	21	Cutaneous stimulation	60 minutes	MAS	-1.0*	0.75

Table S1 A sample of related work used for our power analysis with Modified Ashworth Scale (MAS) as an outcome measure. # indicates the number of participants in the treatment group. MAS scores are on a scale of 0-5. MAS is reported for the finger flexors [3], soleus [1], and rectus femoris [2]. Δ indicates the average change in MAS from baseline to the measure immediately following stimulation (or during stimulation for Murillo et al.). SD indicates the standard deviation. * indicates statistically significant change from baseline.

2 RESULTS – ADDITIONAL DATA

Additional graphs and raw data are shown in Figures S3 and S4, and Table S1. The figures show the average value at each time to illustrate how the spastic hypertonia changed over time. Table S1 provides difference from baseline ($\tau 0$) at each subsequent time point.

Modified Ashworth Scale: The difference from baseline at $\tau 1$ (during stimulation) was not significant for agonist muscle stimulation (Wilcoxon Signed-Rank test: Z = 1.22, p = 0.11) or antagonist muscle stimulation (Wilcoxon Signed-Rank test: Z = 0.74, p = 0.23). The average difference for the control condition was 0.

MTS Ratings: The difference from baseline at $\tau 1$ (during stimulation) was not significant for agonist muscle stimulation (Wilcoxon Signed-Rank test: Z = 1.29, p = 0.098) or antagonist muscle stimulation (Wilcoxon Signed-Rank test: Z = 0.84, p = 0.20). The average difference for the control condition was 0.

MTS Angle: The MTS angles reported here are calculated as the difference between maximum passive extension (how far the fingers can be stretched open slowly) and the spastic catch angle (the angle at which the spastic muscles halt a rapid stretch of the fingers). This angle is part of the standard MTS procedure (R2-R1). The difference from baseline at $\tau 1$ (during stimulation) was not significant for agonist muscle stimulation (t(13) = -1.66, p = 0.060) or antagonist muscle stimulation (t(13) = -2.38, p = 0.017).

Isometric Force Pillow: The measure recorded a difference in average involuntary finger flexion force between baseline and during stimulation ($\tau 1$) for all conditions: agonist muscle stimulation (Avg. = -0.92 mmHg), antagonist muscle stimulation (Avg. = -0.93 mmHg), finger cutaneous stimulation (Avg. = -1.25 mmHg), control (Avg. = -0.77 mmHg). For example, ranging from +1 mmHg to -8 mmHg for the finger cutaneous stimulation condition. At $\tau 3$ after the gripping exercise, mean values increased past baseline

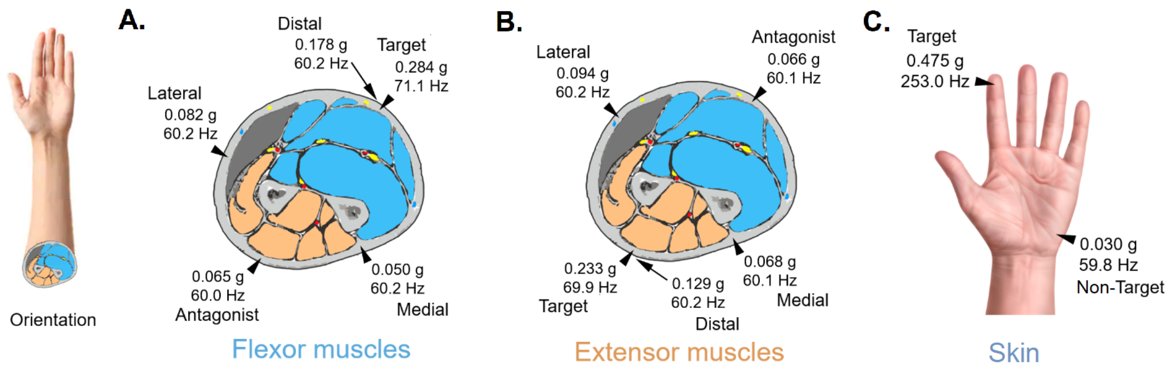

Figure S1. Validation data for the apparatus when mounted on an arm, showing the low level of transmission at non-target regions around the targeted stimulation zone. A. Spastic (flexor) muscle stimulation. B. Antagonist (extensor) muscle stimulation. C. Cutaneous stimulation.

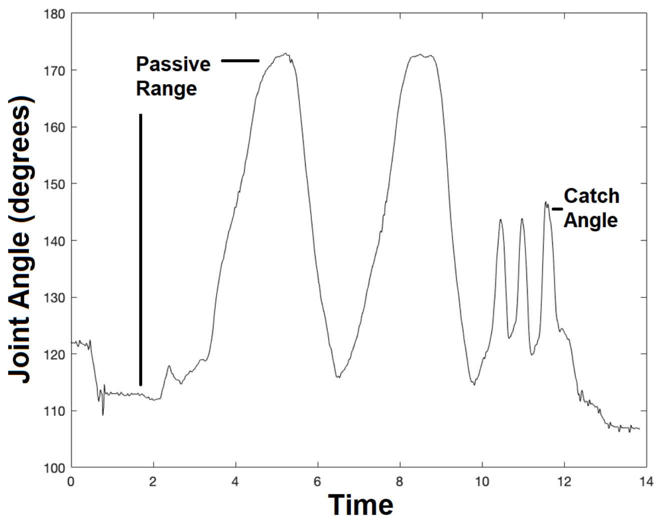

Figure S2. Sample recording of passive range of motion and catch angle using 3D motion capture to track the joint angle. The passive range (approximately 110 degrees to 170 degrees in this example) is measured as the average of two slow excursions, and the catch angle (approximately 145 degrees in this example) is measured as the average of three quick excursions.

values for the antagonist stimulation condition and control condition, but remained below baseline for agonist muscle stimulation (-1.29 mmHg) and finger cutaneous stimulation (-1.02 mmHg).

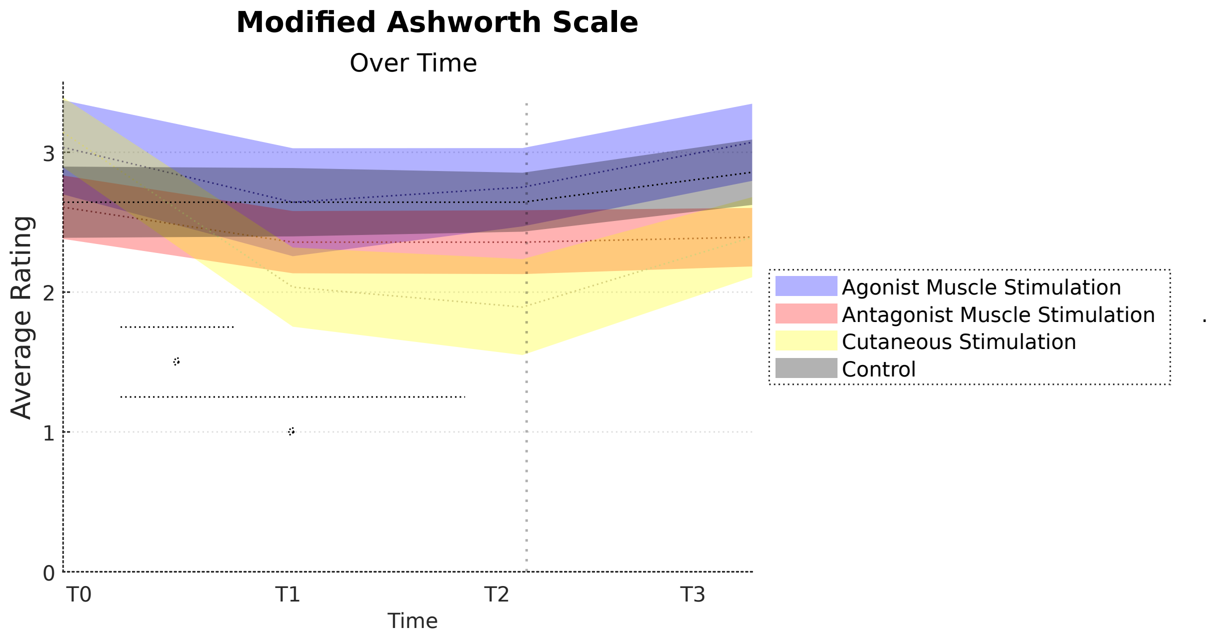

Figure S3. Average Modified Ashworth scale ratings at each time point for all conditions. Lower ratings indicate lower spastic hypertonia. The vertical dotted line indicates the gripping exercise between $\tau2$ and $\tau3$, after which ratings are expected to increase. The shaded region indicates standard error. Asterisks indicate the significance difference in the cutaneous stimulation condition between time points. MAS ratings are presented on a scale of 0-5.

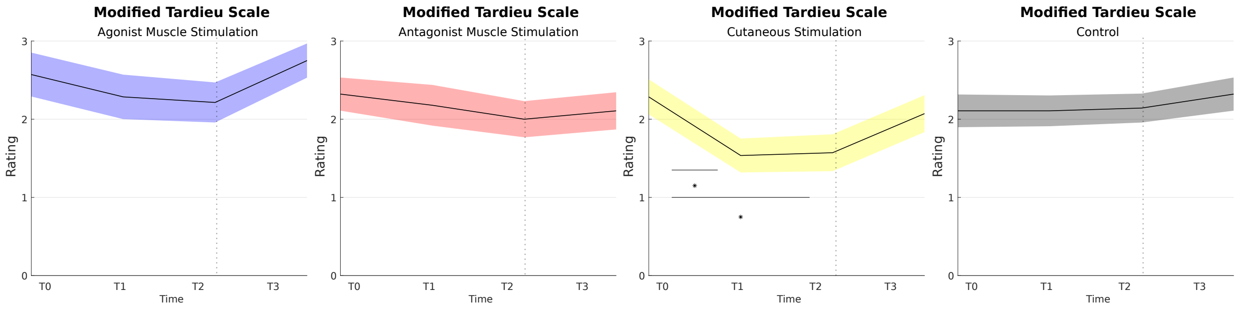

Figure S4. Average Modified Tardieu scale ratings at each time point for all conditions. The shaded region indicates standard error. Vertical dotted lines indicate the gripping exercise between $\tau2$ and $\tau3$, after which ratings are expected to increase. Lower ratings indicate lower spastic hypertonia.

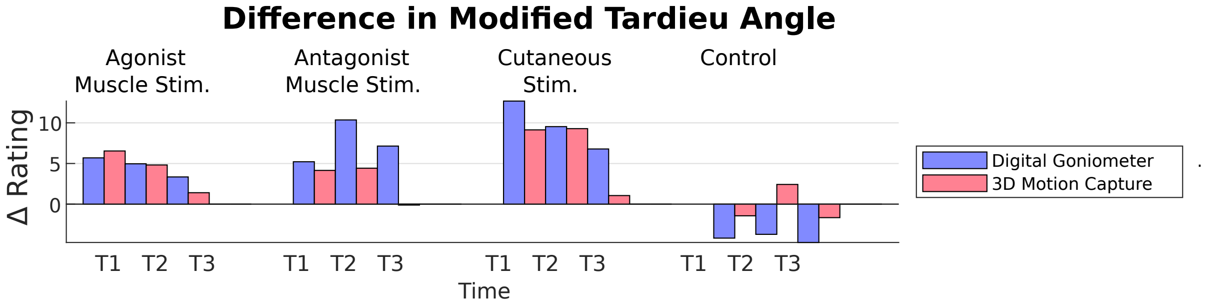

Figure S5. The average difference in Modified Tardieu angle (in degrees) for all time points and all conditions.

		MAS Rating		MTS Rating		MTS Angle	
		Avg. Δ	SD	Avg. Δ	SD	Avg. Δ	SD
Agonist Muscle Stimulation	During Stimulation (τ1)	-0.39	0.96	-0.29	0.72	-5.68	12.79
	After Rest (τ2)	-0.29	0.72	-0.36	0.72	-4.93	14.26
	After Gripping Exercise (τ3)	-0.04	0.93	+0.18	0.88	-3.35	14.18
Antagonist Muscle Stimulation	During Stimulation	-0.25	0.86	-0.14	0.61	-5.21	7.88
	After Rest	-0.25	0.98	-0.32	0.67	-10.36	15.69
	After Gripping Exercise	-0.21	0.65	-0.21	0.56	-7.14	15.66
Finger Cutaneous Stimulation	During Stimulation	-1.11	0.84	-0.75	0.65	-12.68	13.09
	After Rest	-1.25	0.94	-0.71	0.67	-9.54	11.96
	After Gripping Exercise	-0.75	1.01	-0.21	0.56	-6.79	17.54
Control	During Stimulation	0	0.56	0	0.27	-4.18	14.88
	After Rest	0	0.56	+0.04	0.52	+3.71	11.9
	After Gripping Exercise	+0.21	0.45	+0.21	0.41	+4.71	12.7

Table S1. Average difference in Modified Ashworth Scale (MAS) ratings and Modified Tardieu (MTS) values at each time point for all conditions. Avg. Δ is the average change from baseline. SD is standard deviation. MAS ratings are presented on a scale of 0-5. Negative numbers represent a reduction in spastic hypertonia. The score for each participant at a given time point was calculated by averaging their PIP and MCP values.

REFERENCES

[1]H.-y. Cho, T. S. In, K. H. Cho, and C. H. Song. A single trial of transcutaneous electrical nerve stimulation (TENS) improves spasticity and balance in patients with chronic stroke. *Journal of Experimental Medicine*, 229(3):187–193, 2013.

[2]N. Murillo, H. Kumru, J. Vidal-Samso, J. Benito, J. Medina, X. Navarro, and J. Valls-Sole. Decrease of spasticity with muscle vibration in patients with spinal cord injury. *Clinical Neurophysiology*, 122(6):1183–1189, 2011.

[3]T. Noma, S. Matsumoto, S. Etoh, M. Shimodozono, and K. Kawahira. Anti-spastic effects of the direct application of vibratory stimuli to the spastic muscles of hemiplegic limbs in post-stroke patients. *Brain Injury*, 23(7-8):623–631, 2009.
